# Medical Lectures

**Published:** 1844-09

**Authors:** 


MEDICAL LECTURES.

The lectures in the *Louisville Summer School of Medicine* are resumed to-day, and will be continued without interruption till the last of October. Clinical lectures will be delivered on each Wednesday and Saturday, at the Louisville Marine Hospital, and on every other day in the week, Sabbaths excepted, three lectures on the various branches of the profession will be delivered in the rooms of the Medical Institute. The charge for this course of lectures is $15. The Hospital presents at this time much that is interesting to Students of medicine. Dr. Bayless will open the dissecting rooms the first of October, and the most desirable opportunities will be afforded for prosecuting practical anatomy.

